# Dissociated lower limb muscle involvement in amyotrophic lateral sclerosis and its differential diagnosis value

**DOI:** 10.1038/s41598-019-54372-y

**Published:** 2019-11-28

**Authors:** Fangfang Hu, Jiaoting Jin, Qiaoyi Chen, Li Kang, Rui Jia, Xing Qin, Xiao Liu, Yonghui Dang, Jingxia Dang

**Affiliations:** 1 Department of Neurology, The First Affiliated Hospital of Xi’an Jiaotong University, 277 West Yanta Road, Xi’an, Shaanxi 710061 China; 2 Department of Environmental Medicine, New York University School of Medicine, New York, NY USA; 3 College of Medicine & Forensics, Xi’an Jiaotong University Health Science Center, 76 West Yanta Road, Xi’an, Shaanxi 710061 China

**Keywords:** Diagnostic markers, Amyotrophic lateral sclerosis

## Abstract

To explore differential diagnosis value of dissociated lower-limb muscle impairment, we performed a retrospective analysis of clinical and electrophysiological features in 141 lower-limb involved ALS patients, 218 normal controls, 67 disease controls, and 32 lumbar spondylosis disease patients. The dissociated lower-limb muscle impairment was quantified by plantar flexion and dorsiflexion strength, compound muscle action potentials ratio of peroneal and tibial nerves (split index, SI) and semi-quantitative scoring scale of denervation potential. Clinical features: the proportion of decreased dorsiflexion was higher than decreased planter flexor strength in lower-limb involved ALS (77.2%vs 38.3%). Electrophysiological features: (1) SI in ALS was the lowest among four groups (Test statistic = 40.57, p < 0.001). (2) Percentage of positive denervation potential was higher in tibialis anterior than gastrocnemius muscle (χ^2^ = 87.12, p < 0.001). ROC curve: the SI cutoff was 0.52 and 0.33 respectively to differentiate ALS from lumbar spondylosis disease and peripheral neuropathy. Lower-limb involved ALS patients exhibited “split leg” phenomenon. The SI value could be used as an electrophysiological marker to differentiate ALS from lumbar spondylosis disease and peripheral neuropathy.

## Introduction

Amyotrophic lateral sclerosis (ALS) is a devastating illness characterized with limb weakness, muscle atrophy, stiffness, and fasciculation^1^. Depending on site of disease onset, ALS is divided into two groups: bulbar-onset and limb-onset ALS, 75% of which are limb-onset^2^. Clinically, muscle weakness and atrophy in limb-onset ALS patients are typically focal, early misdiagnosis of ALS remains a common clinical problem. A retrospective study showed approximately 5% of patients with ALS undergo spinal surgery early in their clinical course, 42% of them underwent lumbar operations^3^. Careful differential diagnosis for ALS is necessary before making decisions about spinal surgery.

Wilbourn^4^ first reported split hand syndrome in upper limb-onset ALS patients, which refer to Abductor pollicis brevis (APB) and first dorsal interosseous (FDI) muscles being predominantly affected by relative sparing of abductor digiti minimi (ADM). Menon P and Park D^5^ further confirmed this phenomena through electrophysiology studies. They revealed that compound muscle action potentials (CMAP) of APB and FDI were significantly lower than CMAP of ADM. The results were presented as split hand index (SI, ${\rm{SI}}=({\rm{CMAPAPB}}\,\times {\rm{CMAPFDI}})\div{\rm{CMAPADM}}$)^5,6^.They found that the “split hand” could be used to differentiate ALS from CSA^5,7^. Recently, Neil reported dissociated lower limb muscle involvement in ALS^8^. This was similar to the phenomenon of split hand syndrome. He found that there was significant asymmetrical wasting of ankle dorsiflexion muscle and plantar flexor muscle, named split leg.

The ankle dorsiflexion weakness may cause foot drop, which is a common clinical manifestation and may be a prominent feature of lower limb-onset ALS patients during the early stages. The most frequent cause of foot drop is peroneal neuropathy at the fibular head. However in some ALS, lumbar spondylosis disease and peripheral neuropathy patients^9^ have also been reported to experience foot drop. Nerve conduction block is the typical manifestation of peroneal neuropathy in electrophysiological examination^10^. But there is a paucity of electrophysiological studies to differentiate lower limb-onset ALS from lumbar spondylosis disease and peripheral neuropathy.

Our study aims to analyze patterns of ankle dorsiflexor and plantar flexor muscles involvement in lower limb involved ALS, as well as potential diagnostic values in distinguishing ALS from lumbar spondylosis disease and peripheral neuropathy.

## Subjects and Methods

### Subjects

303 ALS patients with probable or definite ALS met the revised Awaji diagnostic criteria^11^ and were recruited from March 2011 to July 2018. The participants’ male to female ration was 1.29, and the mean age was 54.63 ± 8.70 years. Among them, 141 lower limb involved ALS patients were included in our study. Lower limb was considered to be involved^8^ if any of following criteria were met: (1) complaint of weakness or muscle atrophy in the lower extremities, (2) or weakened muscle strength detected from lower limb clinical examination, (3) or points lost on lower limb tasks of the ALSFRS-R (items 7, 8 and 9), (4) or evidence of denervation potential and reduced recruitment from lower limb needle electromyography (EMG) examination.

Control data for nerve conduction studies were obtained from 218 age- and gender-matched normal subjects (male-to-female ratio: 1.53, mean age: 53.96 ± 13.15 years), 64 in disease control group (male-to-female: 2.05, mean age: 57.27 ± 11.00 years) including 30 type-2 diabetic peripheral neuropathy, 24 Guillain-Barrés syndrome (GBS), 9 chronic inflammatory demyelinating polyneuropathy (CIDP) and 4 Charcot-Marie-Tooth disease (CMT), and 32 lumbar spondylosis disease group (male-to-female 2.1, mean age 55.35 ± 10.04 years).

The criteria for lumbar spondylosis disease diagnosis include: (1) unilateral or bilateral weakness of the distal lower limbs accompanied by atrophy of distal muscle; (2) minimal or absence of sensory deficit in the lower limbs; (3) absence of ankle-jerks; (4) presence of lumbar spondylosis supported by MRI lumbar imaging. The exclusion criteria includes: (1) patients with unobtainable CMAP amplitude; (2) previous history of trauma in lower limbs or focal compressive neuropathy, such as peroneal neuropathy at the fibula head; (3) previously diagnosed peripheral polyneuropathy, (4) previous history of an autoimmune-related disease; (5) lumbar plexus lesions; (6) spinal cord tumor or anatomic anomaly in the lumbar vertebrae.

We stated that informed consent was obtained from all subjects. This study was approved by the Research Ethics Committee of the First Affiliated Hospital of Xi’an Jiaotong University. We confirmed that our study was performed in accordance with relevant guidelines and regulations.

### Clinical assessment

Clinical assessments for each patient were recorded prior to electrophysiology examination. Motor functional status was assessed using the revised ALS Functional Rating Scale (ALSFRS-R)^12^. The onset to diagnosis interval (ODI), duration of lower limb involvement, indicated by the time from onset of lower limb weakness to diagnosis, region of clinical onset, as well as symptomatic body regions at the time clinical reviews were recorded. Muscle strength was assessed using the standard Medical Research Council (MRC) rating scale^8^. Ankle dorsiflexion and plantar flexion were graded for each patient.

### Electrophysiology studies

Nerve conduction studies (NCS) were carried out at skin temperatures above 32 °C^13^ using an electro-diagnostic device and software (EDX EMG/evoked potential equipment, Nicolet, America). The surface recording electrode was placed over the belly of extensor digitorum brevis and hallucal abductor muscle, which was confirmed by contraction, in response to peroneal nerve and tibial nerve respectively. Reference electrode was place 2–3 cm away from the recording electrode. In order to ensure that the location of the recording electrode was accurate and get the maximum amplitude of CMAP, we slightly adjusted the position of the recording electrode repeatedly. At the same time, The peroneal and tibial nerves were stimulated at the ankle and medial malleolus with supramaximal stimuli (1.5-fold maximal intensity). The amplitude of CMAP was recorded from baseline to negative peak^14^. The split leg index (SI) was calculated using the following equation:

$${\rm{SI}}={\rm{CMAPDF}}\div{{\rm{CMAPPF}}}^{8}$$

Concentric needle EMG examination was performed on tibialis anterior and gastrocnemius muscles to identify neurogenic damage (spontaneous muscle fiber activity, giant /polyphasic MUPs and reduced recruitment). The spontaneous muscle fiber activity (fibrillation potential and positive sharp wave) was recorded by semi-quantitative scoring scale (0–4)^15^. We thought neurogenic damage existed if there was fibrillation potential and/ or positive sharp wave in two points.

Nerve conduction studies and needle EMG were conducted for ALS and lumbar spondylosis disease patients. Others subjects only underwent nerve conduction examination. All of electrophysiology examination was performed by an electrophysiologist with nine years of experience. We must check and confirm the electrophysiological results were consistent with clinical symptoms.

### Statistical analysis

**Figure 1:**
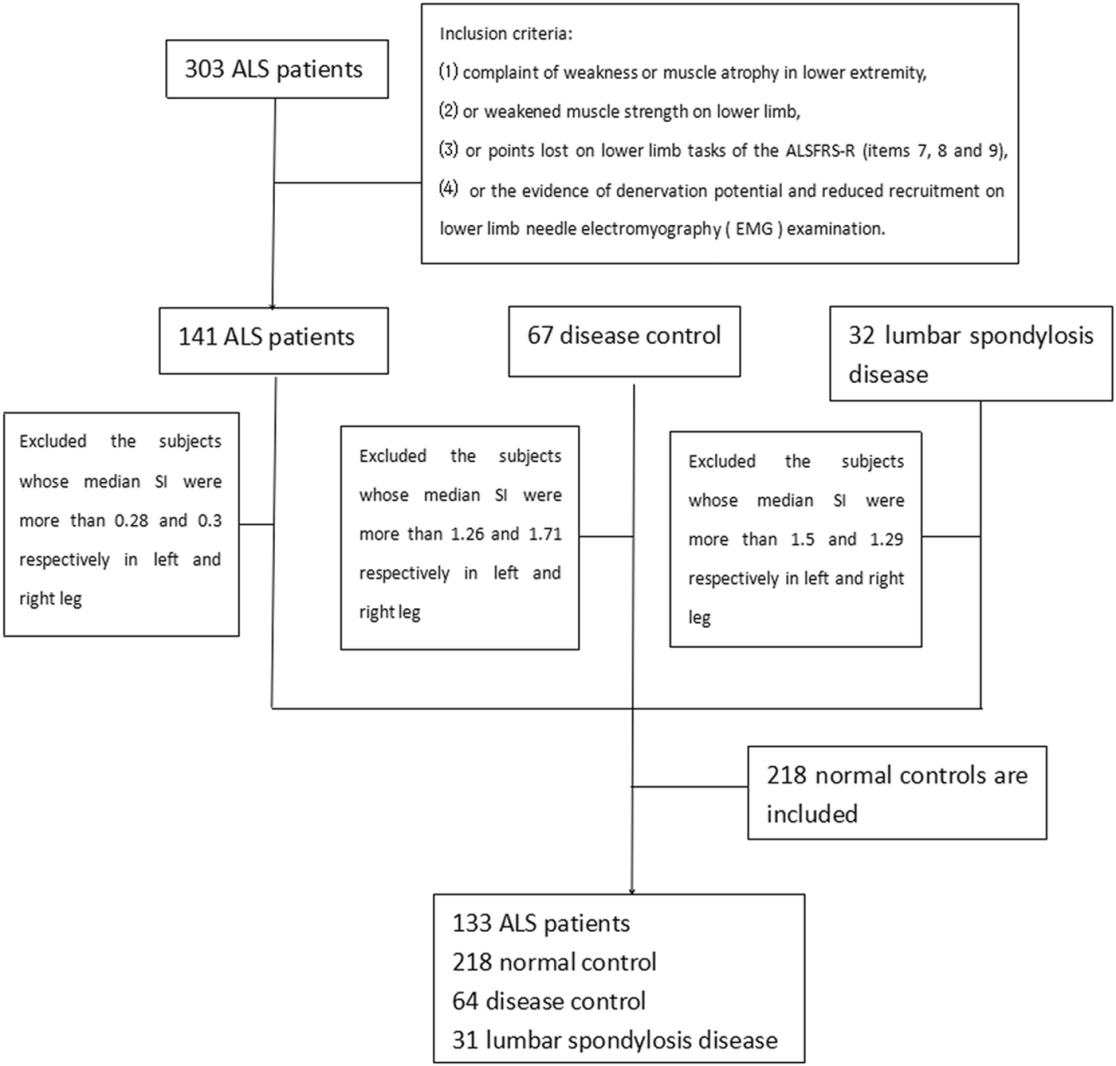
Flowchart of this study.

Baseline analyses included all patients. The mean of SI was analyzed if bilateral lower extremities were involved, the SI of involved lower limb was analyzed if only one lower extremity was involved. The Kolmogorov-Smirnov test showed that duration to lower limb, ALSFRS-R, ODI, and SI did not conform to normal distribution (p < 0.05), the values were shown as median. The values for age-onset, amplitude of tibial and peroneal nerve were shown to conform to normal distribution (p > 0.05), the values were shown as mean ± SD. Box figure illustrates outliers of SI, the subjects with SI 3-folds above the median were excluded (Fig. 1).

The general multivariate linear models were performed to determine the differences in SI among the four groups (ALS, disease control group, lumbar spondylosis and normal control group), while controlling for age. All analyses were Bonferroni-corrected for multiple comparisons. Spearman correlation was used to correlate SI and clinical variables at baseline. The Pearson Chi-square test was performed to analyze the difference in denervation potential between tibialis anterior and gastrocnemius muscle. ROC curve was performed to identify the predicting values of SI. p < 0.05 was considered statistically significant.

## Results

Among 133 ALS patients, 6.77% (9) had bulbar onset, 56.39% (75) had upper extremity onset, and 36.84% (49) had lower extremity onset. The mean onset to diagnosis interval (ODI) was 14.70 ± 9.83months (range 2–54 months). The mean ALSFRS-R score was 38.02 ± 6.11, and the mean sub-ALSFRS-R score in the lower extremities was 7.73 ± 2.59, suggesting a mild-to- moderate degree of disability.

### Clinical characteristics

236 lower extremities were affected in 133 ALS patients, 78% (67) of them involved the bilateral lower extremities, and 22% (66) involved one lower extremity. The median ankle dorsiflexion strength was 4/5, plantar flexion strength was 5/5. In terms of clinical detectable weakness in lower extremity, 70.8% (167) had clinical detectable limb weakness and EMG positive (clinical weakness group), 29.2% (69) showed only EMG positive without limb weakness (EMG positive group).

In clinical weakness group, the proportion of decreased dorsiflexion was higher than decreased planter flexor strength in ALS patients with lower limb involvement (77.2%vs 38.3%). Ankle dorsiflexion and plantar flexion strength were found equal on the MRC rating scale in 35.9% of limbs. Ankle dorsiflexion was weaker than plantar flexion in 61.1% of limbs. Dorsiflexion was stronger than plantar flexion in 1.8% of limbs. The median ratio of ankle dorsiflexion to ankle plantar flexion MRC scores was 0.8 (range 0.0–2.0). (Table 1).

**Table 1:** Clinical features of ALS patients.

	Clinical weakness group (n = 167)	EMG positive group (n = 69)	All patients (n = 236)
Age	54.51 ± 8.27	57.19 ± 7.62	55.13 ± 8.17
Gender			
Male/female	1.30	1.62	1.38
Lower extremity involved time (M)	6.5 (1~38)	NA	6.5 (1~38)
ALSFRS-R	37.75 ± 6.37	38.96 ± 6.18	38.03 ± 6.32
Sub-ALSFRS-R score in lower extremity	6.88 ± 2.34	9.56 ± 2.31	7.50 ± 2.59
Amplitude in tibial nerve (mv)	7.64 ± 3.71	10.00 ± 3.66	8.37 ± 3.85
Amplitude in peroneal nerve (mv)	2.05 ± 1.69	2.99 ± 2.07	2.35 ± 1.86
Affected lower extremity			
With decreased dorsiflexion strength (<5/5)	77.2% (129)	15.9% (11)	59.3% (140)
With decreased plantar flexion strength (<5/5)	38.3% (103)	5.8% (4)	45.3% (107)
Median MRC score (median)			
Dorsiflexion strength	4/5	5/5	4/5
Plantar flexion strength	5/5	5/5	5/5
MRC score comparison (involved lower limbs)			
Dorsiflexion <plantar flexion	62.3% (104)	13% (9)	47.9% (113)
Dorsiflexion = plantar flexion	35.9% (60)	84.1% (58)	50% (118)
Dorsiflexion >plantar flexion	1.8% (3)	2.9% (2)	2.1% (5)

In EMG positive group, dorsiflexion and plantar flexion strength were found equal in 84.1% of lower extremity. The median ratio of ankle dorsiflexion to ankle plantar flexion MRC scores was 1.0 (range 0.6–1.33). (Table 1).

### Neurophysiological characteristics

**Figure 2:**
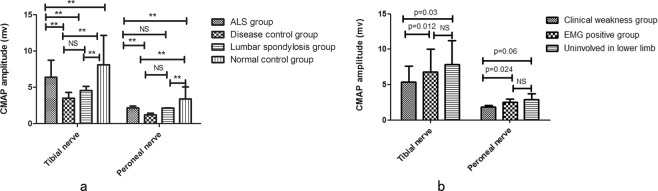
CMAP_DF_ and CMAP_PF_ were analyzed in different groups: (**a**) CMAP_DF_ and CMAP_PF_ were significantly more reduced in the disease control group compared with ALS group (p = 0.006, p < 0.001), no difference in CMAP_DF_ was shown between ALS and lumbar spondylosis group (p = 1.000), CMAP_PF_ was more reduced in lumbar spondylosis group than ALS group (p < 0.001), and no difference in CMAP_PF_ was detected between disease control group and lumbar spondylosis group (p = 1.000). (**b**) CMAP_DF_ and CMAP_PF_ were more strongly reduced in clinical weakness group than EMG positive and uninvolved in lower limb groups. ** indicates p < 0.05, NS indicates lack of significant difference.

The waveform of 29 peroneal nerves and 1 tibial nerve were not elicited in ALS patients. The differences of CMAP_DF_, CMAP_PF_ and SI were analyzed using age as covariate in the general multivariate linear models. CMAP_DF_ and CMAP_PF_ showed significant differences among ALS group, normal control group, disease control group and lumbar spondylosis group (CMAP_DF_ 2.42 ± 1.9 vs 5.04 ± 1.708 vs 1.43 ± 0.95 vs 2.11 ± 2.17 p < 0.001, CMAP_PF_ 8.73 ± 4.02 vs 12.14 ± 4.06 vs 4.29 ± 2.67 vs 5.12 ± 3.99, p < 0.001 Fig. 2a). In the ALS group, CMAP_DF_ and CMAP_PF_ had significant differences among clinical weakness group, EMG positive group and uninvolved in lower limb group (CMAP_DF_ 2.05 ± 1.69 vs 3.00 ± 2.07 vs 3.72 ± 2.04 p = 0.001, CMAP_PF_ 7.64 ± 3.71 vs 10.00 ± 3.66 vs 11.23 ± 4.43 p < 0.001 Fig. 2b).

**Figure 3:**
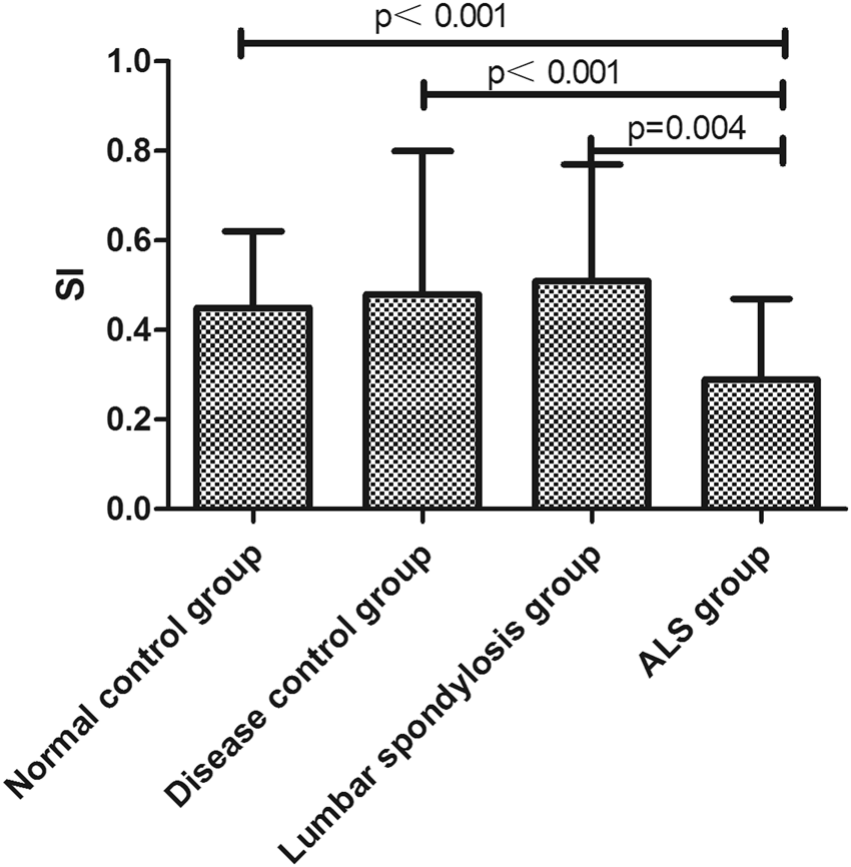
The comparison of SI among ALS group, normal group, disease control group and lumbar spondylosis group.

SI showed significant differences among ALS, normal control, disease control and lumbar spondylosis groups (SI: 0.32 ± 0.20 vs 0.45 ± 0.17 vs 0.49 ± 0.32 vs 0.47 ± 0.30 p < 0.001). Further paired comparisons, SI was found to be the lowest in ALS patients and highest in disease group or lumbar spondylosis group. However there were no difference between disease group, lumbar spondylosis group and normal control group (Fig. 3). SI was lower in the clinical weakness group compared with EMG positive and uninvolved in lower limb groups, but the difference was insignificant (0.30 ± 0.22 vs 0.33 ± 0.23 vs 0.34 ± 0.17, Test statistic = 1.14, p = 0.56).

Correlations between SI and clinical variables were analyzed. Negative correlation was found between SI and lower limb involved time (spearman r = −0.221, p = 0.014). There were no correlations between SI and sub-ALSFRS-R score in lower limb (p = 0.47), ALSFRS-R (p = 0.27), dorsiflexion and plantar strength (p = 0.16, 0.57). There were strong correlations between SI and CMAP amplitude in peroneal and tibial nerves (Spearman r = 0.61 p < 0.001, Spearman r = −0.34 p < 0.001).

**Figure 4:**
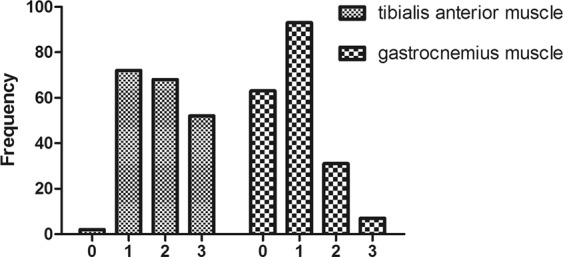
Denervation potential in tibialis anterior and gastrocnemmius muscle.

Concentric needle EMG was examined in 194 tibialis anterior muscles and 198 gastrocnemius muscles. 75% (145) of lower extremities had more denervation potential in tibialis anterior muscle than gastrocnemius muscle. 22% (42) of lower extremities had the same amount of denervation potentials in tibialis anterior muscles and gastrocnemius muscles. Only 4% (7) with denervation potentials had less in tibialis anterior muscles than in gastrocnemius muscles. The amount of denervation potential was obviously different between tibialis anterior and gastrocnemius muscle (χ^2^ = 87.12, p < 0.001) (Table 2). Positive rate of denervation potentials was much higher in tibialis anterior muscles than gastrocnemius muscles (Fig. 4).

**Table 2:** Amount of denervation potential in tibialis anterior and gastrocnemmius muscle.

	Amount of denervation potential				
	n	0	1	2	3
Tibialis anterior muscle	220	3.1% (7)	35.0% (77)	36.8% (81)	25.0% (55)
Gastrocnemius muscle	197	32.5% (64)	47.2% (93)	16.2% (32)	4.0% (8)

### Cut-off value for parameters predicting ALS

**Figure 5:**
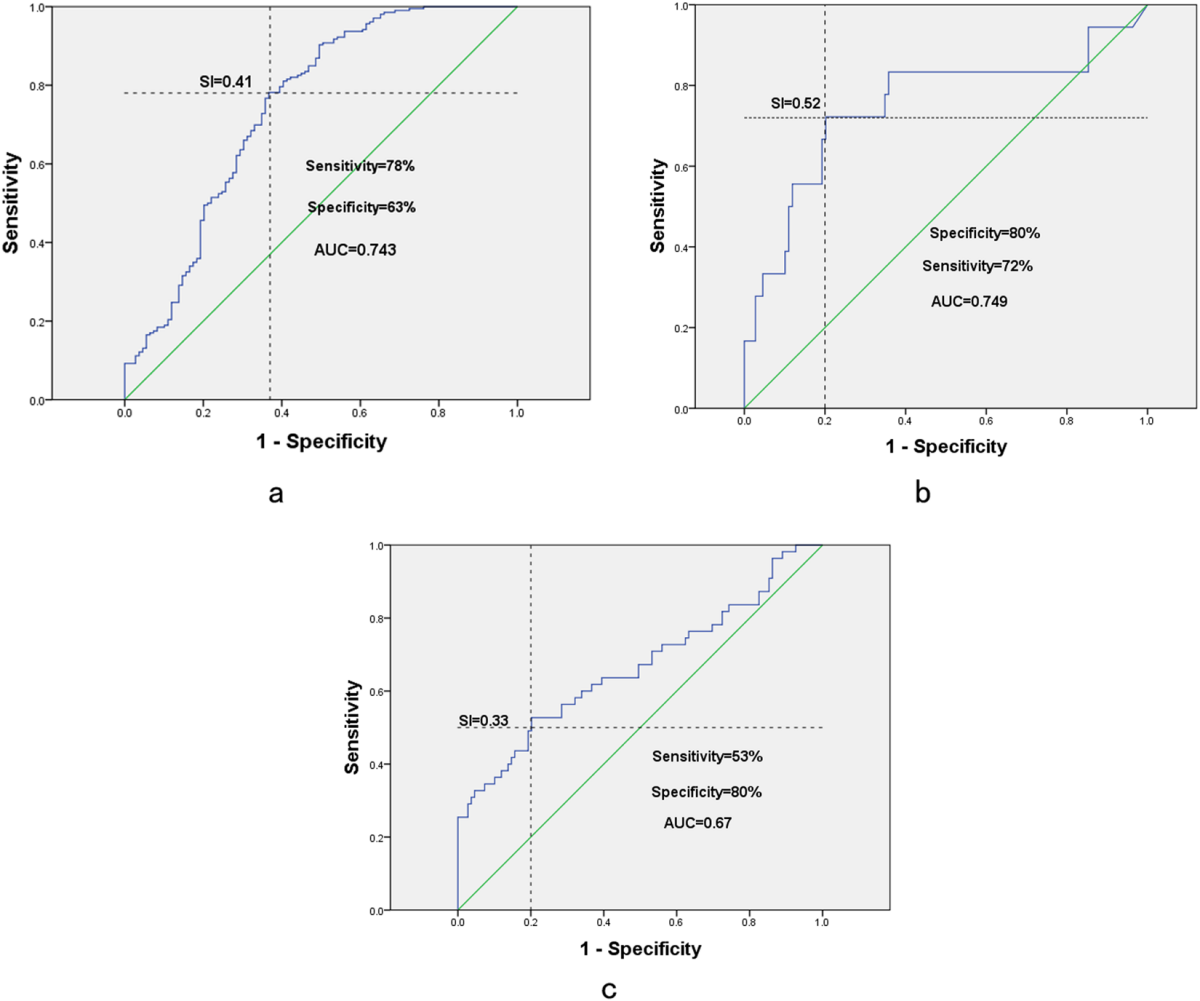
The predictive value of median SI: (**a**) ALS group vs normal control group, (**b**) ALS group vs lumbar spondylosis group, and (**c**) ALS group vs disease control group.

The SI in ALS patients was significantly different from those of other three groups (normal group, disease control group and lumbar spondylosis group). We further analyzed the data to examine the predictive value of median SI using ROC curve. The cut-off value for differential diagnosis of ALS vs normal control group was 0.41 (AUC = 0.743, 95% CI 0.682–0.805), for differential diagnosis of ALS vs lumbar spondylosis group was 0.52 (AUC = 0.749, 95% CI 0.602–0.896), and for differential diagnosis of ALS vs disease control group was 0.33 (AUC = 0.666, 95% CI 0.572–0.760). (Fig. 5).

## Discussion

Many studies have found that some muscles seem to be preferentially affected in ALS patients. There already have some studies about the split hand in ALS patients. However, only one study has found this phenomenon in lower limbs named split leg in ALS patients, which indicated that more patients had weaker dorsiflexion compared with plantar flexion in clinical examination, but in electrophysiological results, the CMAP_PF_ were reduced more greatly compare with CMAP_DF_^8^. The clinical and electrophysiological results was opposite.

In our study, we found the dorsiflexion was preferentially affected than plantar flexion in clinical and electrophysiological results. The proportion of decreased dorsiflexion was higher than decreased planter flexor strength in lower-limb involved ALS. The phenomenon was prominent in ALS patients with clinical detectable lower limb weakness. In addition to the muscle strength assessment, we also used the results of needle EMG to evaluate Neil’s point. The EMG results showed that positive rate and amount of denervation potentials was much higher in tibialis anterior muscle than gastrocnemius muscle. It further confirmed that ankle dorsiflexion muscle was preferentially affected than plantar flexion muscle in lower limb-onset ALS.

The dissociated involvement of distal lower limb muscles was termed as “split leg” phenomenon, which was expressed by SI. However, Our electrophysiological results regarding SI were opposite with Neil G’ results^8^. Neil G showed that the median SI of ALS was significant increased compared with normal controls, suggesting dissociated involvement of lower limb with much more eminent reduction of CMAP_PF_ amplitude than CMAP_DF_ amplitude. In our study, the median SI of ALS was significantly reduced when compared to normal control, disease control and lumbar spondylosis group, which indicated dissociated muscle involvement of lower limb with greater reduction of CMAP_DF_ amplitude than CMAP_PF_ amplitude. We speculated it was caused by two reasons, (1) different target recording muscles. In Neial G’ study, the CMAP of peroneal and tibial nerve was recorded respectively from tibial anterior muscle and gastrocnemius muscle. In our study, CMAP of peroneal and tibial nerve was recorded from the belly of extensor digitorum brevis muscle and hallucal abductor muscle. ⑵ due to the deep position of the tibial nerve in the popliteal fossa, super-strong stimulation may not excite all nerve fibers to reach the maximum amplitude. That caused significant difference of CMAP amplitude of tibial and peroneal nerve and induced the variation of SI.

SI of ALS patients was the lowest when compared to disease control, lumbar spondylosis and normal control groups. It had no differences among disease control, lumbar spondylosis and normal control groups. This indicated that phenomenon of CMAP_DF_ amplitude reduced greater than CMAP_PF_ amplitude mainly occurred in ALS group, the reduction of CMAP_PF_ amplitude and CMAP_DF_ amplitude was found equal in lumbar spondylosis and peripheral neuropathy groups. Reflecting both tibial nerve innervated muscles and peroneal nerve innervated muscles were affected simultaneously. This point was very useful for the differential diagnosis of ALS. In clinical practice, the lower limb-onset ALS was easily misdiagnosed as lumbar spondylosis, especially for non-neuromuscular neurologists. Further ROC analysis showed SI ≦ 0.52 was able to identify ALS with 80% sensitivity and 72% specificity. There were no differences among clinical weakness group, EMG involvement and uninvolved in lower limb groups, it may be caused by only 30 (11.27%) uninvolved lower limb or maybe SI was an earlier feature than EMG in ALS patients.

SI had no correlations with sub-ALSFRS-R score in lower limb, ALSFRS-R, dorsiflexion and plantar flexion strength. But SI had negative correlation with lower limb involved time and CMAP_PF_ amplitude, positive correlation with CMAP_DF_ amplitude. This suggested that SI value was affected by lower limb involved time, CMAP_PF_ and CMAP_DF_ amplitude, not affected by the degree of disability in lower limb. And according to correlation coefficients, CMAP_DF_ amplitude had seriously influence on SI value (0.609 vs −0.343).

About the mechanism of split leg phenomenon, we assume it has the same origin as split hand, (1) primary corticomotoneuronal influence^16^, foot drop may be a prominent feature of corticospinal tract injury, the density and nature of the cortical motoneuronal projections to dorsiflexor and plantar flexor muscles may be contributed to the pathogenesis of the split leg phenomenon; (2) physical activity influence, lower limb activity generally involves tonic activation of the glutei, vasti and plantar flexor muscles while standing and walking^17^, but the correlation was more significant between score of sub-ALSFRS-R in lower limb and ankle dorsiflexion strength than ankle plantar flexion strength (Spearman’s r = 0.523 vs 0.4, p = 0.000, 0.000), so the dorsiflexion muscle may be suffer from greater oxidative stress, resulting in preferential degeneration.

From this study, we concluded that in ALS patients with lower limb involvement, there were greater involvement of ankle dorsiflexion muscle than plantar flexor muscle, which was verified by clinical examination, nerve conduction and EMG. SI could be used as a biomarker to differentiate ALS patients with lower limb involvement from lumbar spondylosis. We hope the split phenomenon could help non-neuromuscular neurologists be vigilant when looking at a patient with focal weakness and atrophy of the lower extremities.

## Supplementary information

Supplementary information

## Data Availability

The datasets generated and analysed during the study are included in its Supplementary Information Files.
